# Dosimetric Comparisons of Simulation Techniques for Left-Sided Breast Cancer in the COVID-19 Era: Techniques to Reduce Viral Transmission and Respect the Therapeutic Ratio

**DOI:** 10.7759/cureus.13354

**Published:** 2021-02-15

**Authors:** James M Taylor, Andrew Song, Kamila Nowak, Tu Dan, Brittany Simone, Amy Harrison, Laura Doyle, Virginia Lockamy, Pramila Anne, Nicole Simone

**Affiliations:** 1 Radiation Oncology, Sidney Kimmel Cancer Center at Thomas Jefferson University Hospital, Philadelphia, USA

**Keywords:** breast cancer research, adjuvant radiation therapy, patient dosimetry, photon dosimetry, prone positioning, deep-inspiration breath-hold, covid-19

## Abstract

Background

The COVID-19 pandemic challenges our ability to safely treat breast cancer patients and requires revisiting current techniques to evaluate optimal strategies. Potential long-term sequelae of breast radiation have been addressed by deep inspiration breath-hold (DIBH), prone positioning, and four-dimensional computed tomography (4DCT) average intensity projection (AveIP)-based planning techniques. Dosimetric comparisons to determine the optimal technique to minimize the normal tissue dose for left-sided breast cancers have not been performed.

Methods

Ten patients with left-sided, early-stage breast cancer undergoing whole breast radiation were simulated in the prone position, supine with DIBH, and with a free-breathing 4DCT scan. The target and organs at risk (OAR) contours were delineated in all scans. Target volume coverage and OAR doses were assessed. One-way analysis of variance (ANOVA) and Kruskal-Wallis one-way ANOVA were used to detect differences in dosimetric parameters among the different treatment plans. Significance was set as p < 0.05.

Results

We demonstrate differences in heart and lung dose by the simulation technique. The mean heart doses in the prone, DIBH, and AveIP plans were 129 cGy, 154 cGy, and 262 cGy, respectively (p=0.02). The lung V20 in the prone, DIBH, and AveIP groups was 0.5%, 10.3% and 9.5%, respectively (p <0.001). Regardless of technique, lumpectomy planning target volume (PTV) coverage did not differ between the three plans with 95% of the lumpectomy PTV volume covered by 100.4% in prone plans, 98.5% in AveIP plans, and 99.3% in DIBH plans (p=0.7).

Conclusions

Prone positioning provides dosimetric advantages as compared to DIBH. When infection risks are considered as in the current coronavirus disease 2019 (COVID-19) pandemic, prone plans have advantages in reducing the risk of disease transmission. In instances where prone positioning is not feasible, obtaining an AveIP simulation may be useful in more accurately assessing heart and lung toxicity and informing a risk/benefit discussion of DIBH vs free breath-hold techniques.

## Introduction

The ongoing coronavirus disease 2019 (COVID-19) pandemic presents unique challenges to clinical practices for treating cancer patients safely and responsibly. Concerns for minimizing risks of transmission amongst patients and providers have become part of the forefront of our daily lives. For breast cancer patients, this has resulted in a paradigm shift of radiation practice guidelines, including deferral of radiation treatment starts, various hypofractionation schedules, or omission of radiation therapy altogether [1-2].

Routine practices have been evaluated to ensure possible COVID-19 transmission is kept to a minimum while still delivering optimal radiation. In this manner, the use of deep inspiratory breath-hold (DIBH) with Active Breathing Control (ABC) devices (Active Breathing Coordinator™, Elekta, Stockholm, Sweden) to provide cardiac dose sparing for early-stage left breast cancer patients has come under scrutiny due to the possibility of COVID-19 spread on shared devices [3]. DIBH utilizing ABC devices has become a ubiquitous method to provide the ability to spare cardiac dose since several dosimetric studies of DIBH and prospective trials show a decrease in the volume of heart receiving radiation [4-7]. Therefore, if centers decide to temporarily avoid using DIBH with ABC, typical cardiac dose constraints may be difficult to achieve. It is important to note that some centers have the capability to use a DIBH technique without ABC but with optical surface based imaging. This technique would have advantages in the current COVID-19 era in limiting radiation dose to the heart and avoiding the risk of shared respiratory devices. However, despite this, not all institutions have access to optical-based surface imaging and must rely on ABC for DIBH treatments.

Dose sparing techniques are important, as the life expectancy of breast cancer survivors has increased, and thus there are more patients potentially at risk for long-term sequelae secondary to radiation. While the data regarding cardiac toxicity secondary to breast radiation is conflicting, several studies suggest a correlation between radiation to the left breast and an increase in the incidence of cardiovascular and pulmonary events [8]. These include an increase in excess lung cancer and cardiovascular mortality with increasing mean heart dose, which has been validated by the Early Breast Cancer Trialists’ Collaborative Group (EBCTCG) 2005 meta-analysis [8-9].

However, in order to minimize the risk of transmissibility between patients sharing respiratory devices, alternative options should now be prioritized. These can include prone positioning and four-dimensional computed tomography (4DCT)-based planning using an average intensity projection (AveIP). Studies using prone radiation techniques, as with ABC techniques, have also shown decreased doses to the heart and lungs [10]. Compared with ABC and prone positioning, AveIP is not a method for reducing the cardiac dose by itself but can be used to more accurately measure the dose to organs of interest (OARs) during free breathing and provide valuable information to clinicians when deciding on treatment methods during the pandemic. While the data for the dosimetric advantages of heart-sparing techniques is abundant, it remains unclear if one technique offers consistently superior benefits for patients with left-sided breast cancer undergoing breast conservation therapy as compared to traditional techniques.

We studied three techniques employed at our institution for CT simulation of early-stage left-sided breast cancer patients who have undergone breast-conserving surgery, including 1) DIBH 2) prone positioning, and 3) 4DCT AveIP-based planning. We compared the dosimetry of these techniques in the setting of whole breast radiation to the left breast to determine which technique provided the greatest decrease in doses to OARs, including heart and lung, to minimize potential toxicity. Further, we also assessed lumpectomy planning target volume (PTV) coverage among all three plans to identify if a discrepancy in target coverage exists.

## Materials and methods

**Figure 1:**
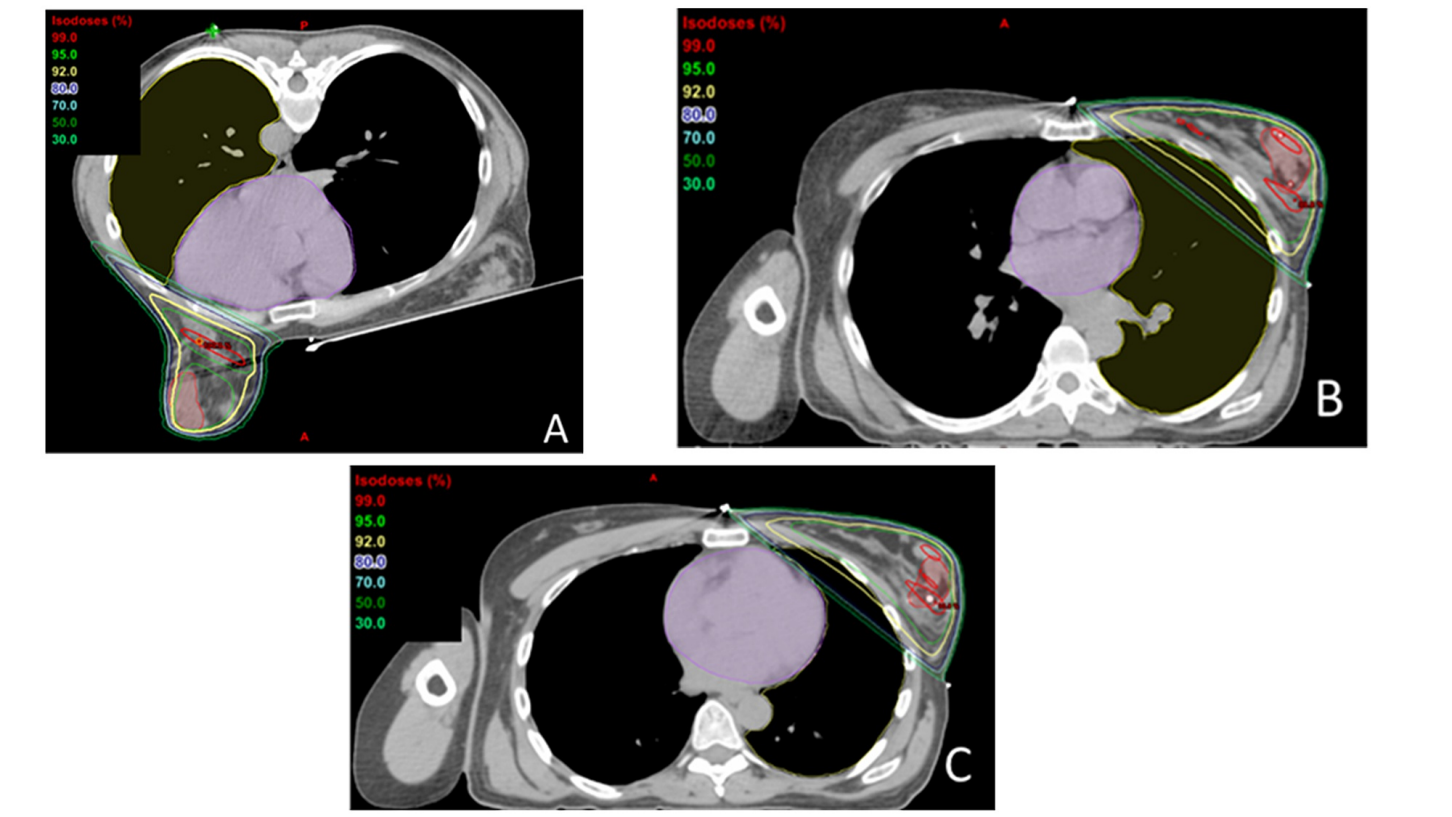
Isodose distribution by the simulation technique A: prone, B: DIBH, C: 4DCT DIBH: deep inspiration breath-hold; 4DCT: four-dimensional computed tomography

In an institutional review board (IRB)-approved study, we retrospectively identified 10 consecutive patients with early-stage, left-sided breast cancer who were to be treated solely with radiation to the breast delivered by tangential fields only and who were planned with three heart-sparing techniques, including supine positioning using either 1) DIBH or 2) 4DCT to evaluate cardiac motion and 3) prone positioning. The anatomic boundaries of the breast tangent borders and lumpectomy scars were marked with a radio-opaque wire prior to CT scan (Figure 1).

To allow for DIBH, patients were positioned supine, with arms overhead, on a breast board at a 10-15 degree angle, connected to the ABC device, and coached by a radiation therapist to hold a deep breath (approximately 70% of total lung capacity) for 20 seconds. Coaching continued until patients could perform deep inspiration breath-hold reliably. A CT scan was then obtained during breath-hold to take advantage of lung hyperinflation to increase the separation between the heart, chest wall, and breast tissue.

A second CT scan was performed in the prone position, with arms overhead, using the Bionix RT-6025 prone breast board (Toledo, Ohio), which uses the advantage of gravity to pull the breast away from the chest wall to decrease the field proximity to the heart and lungs. The wires initially placed on the patient in the supine position were manually appreciated in the prone position by the physician to ensure similar positioning of the breast border.

A 4DCT scan was also performed in the same supine position as DIBH to allow for the tracking of target volumes and OARs during the different phases of respiration to try and determine the anterior-most aspect of the heart. The Varian Real-time Position Management (RPM) system (Palo Alto, California), which utilizes an infrared tracking camera and a reflective marker to follow the patient’s respiratory pattern for the assessment of motion, was used. No abdominal compression was used during the CT scan.

Isocenter localization was performed. Target volumes and OAR volumes, including the heart, lungs, and contralateral breast, were delineated on each of the three scans using the Radiation Therapy Oncology Group (RTOG) breast cancer contouring atlas. For the 4DCT, the AveIP scan was used since it provides an average positional representation of the heart with normal respiration, taking into account the minimum and maximum extent of heart motion through the breathing cycle. The lumpectomy PTV consisted of the lumpectomy CTV with a 1 cm margin. A dose of 50 Gy in 25 fractions was prescribed to the whole breast using opposed tangents, with wedges or ‘field-in-field’ techniques when appropriate. Each plan was individually evaluated to look at target volume coverage and OAR dose and, subsequently, the treating physician determined the appropriate prescription isodose line. Dose-volume histograms (DVHs) were generated and used to compare the dosimetry of the three plans.

Statistical analysis 

One-way analysis of variance (ANOVA) and Kruskal-Wallis ANOVA were used to detect differences in dosimetric parameters among the different treatment plans. For the parameters with significant differences, a paired t-test was used to compare differences among groups. A p-value of ≤0.05 was considered statistically significant.

## Results

Target and OAR volumes

Patient target volumes and OAR volumes were delineated on all three scans by the same physician. A second physician verified volumes. At baseline, when assessing the gross volume, no differences were seen among the three treatment planning techniques for breast, lumpectomy, lumpectomy PTV, and heart volumes.

Both ipsilateral and contralateral lung volumes were consistently larger in the DIBH group, as expected, given the hyperinflation of the lungs during deep inspiration (Table 1).

**Table 1:** Volumetric parameters for organs at risk and planning target volumes

Structure	Mean Volume- Prone	Mean Volume-AveIp	Mean Volume- DIBH	p-value
Breast	1132	915	933	0.64
Lumpectomy	20	24	21	0.80
Lumpectomy PTV	87	95	93	0.93
Ipsilateral Lung	1400	1160	1906	<0.01
Contralateral Lung	1666	1480	2248	<0.01
Heart	595	618	561	0.60

Heart dosimetry

**Figure 2:**
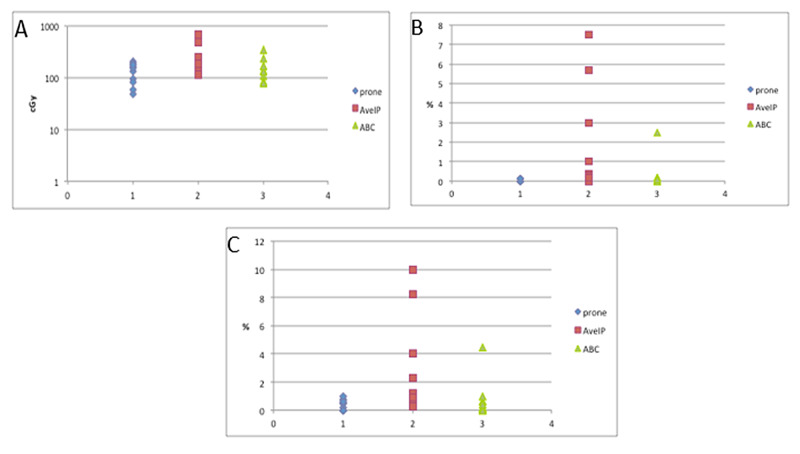
Heart dose by simulation technique A: mean heart dose. B: heart V40. C: heart V20

When comparing the dosimetric parameters related to heart dose, prone and DIBH plans consistently demonstrated lower heart doses as compared to AveIP plans for most of the cardiac parameters examined (Figure 2).

The mean heart doses in the prone, DIBH, and AveIP groups were 129 cGy, 154 cGy, and 262 cGy, respectively (p=0.02) and while the difference in mean heart dose between the DIBH and AveIP plans was statistically significant (p=0.02), a difference was not demonstrated between the prone positioning and DIBH techniques. A similar finding was demonstrated when examining the heart V40, which was 0.01% in the prone group, 0.3% in the DIBH group, and 1.8% in the AveIP group (p=0.02) and heart V20, which was 0.3% in the prone group, 0.7% in the DIBH group, and 2.9% in the AveIP group (p=0.03). The difference in heart V40 and V20 were statistically significantly different between plans utilizing the DIBH and AveIP techniques (p=0.03) and (p=0.02), respectively. Similar to heart mean dose, there was no significant difference between prone positioning and DIBH with respect to heart V40 (p=0.2) or V20 (p=0.1). 

Lung dosimetry

**Figure 3:**
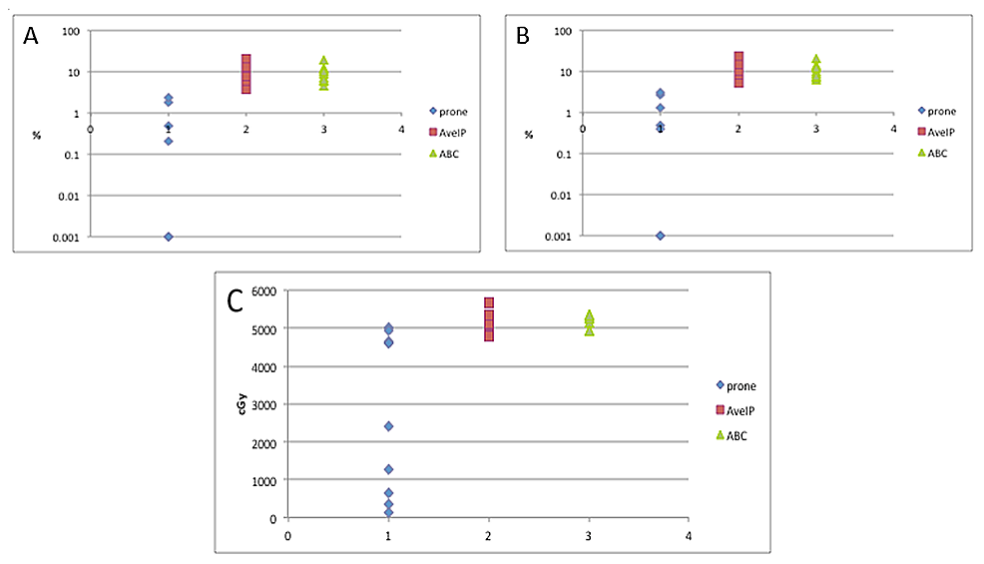
Doses delivered to contralateral and ipsilateral lung by simulation technique A: ipsilateral lung V20. B: ipsilateral lung V10. C: ipsilateral lung maximum dose

Examination of the dosimetric parameters related to lung dose showed significantly decreased doses with prone plans as compared to DIBH and AveIP plans for all pulmonary parameters (Figure 3). The ipsilateral lung V20 was decreased in prone plans with an average of 0.5%, compared with 10.3% for AveIP plans and 9.5% for DIBH plans (p<0.001). When assessing lung V20 between patients treated with AveIP and DIBH plans, no difference was found (p=0.3). Average lung V10 was 0.8% in prone plans, 12.5% for AveIP plans, and 11.3% for DIBH plans (p<0.001). As with lung V20, there were no differences in lung V10 between DIBH and AveIP plans (p=0.2). The ipsilateral lung mean was 68 cGy in the prone group, 596 cGy in the AveIP group, and 553 in the DIBH group (p<0.001). Analysis between the DIBH and AveIP plans did not demonstrate a statistically significant difference in ipsilateral lung mean dose (p=0.2). As with all other lung parameters, no differences between patients treated with the DIBH and AveIP plans were found with respect to the maximum ipsilateral lung dose. Contralateral lung maximum was also decreased in prone plans, where the average maximum dose was 54 cGy, as compared to 164 in AveIP plans and 192 cGy in DIBH plans (p<0.001). The DIBH and AveIP plans did not differ significantly for any of the lung parameters.

Target coverage

**Figure 4:**
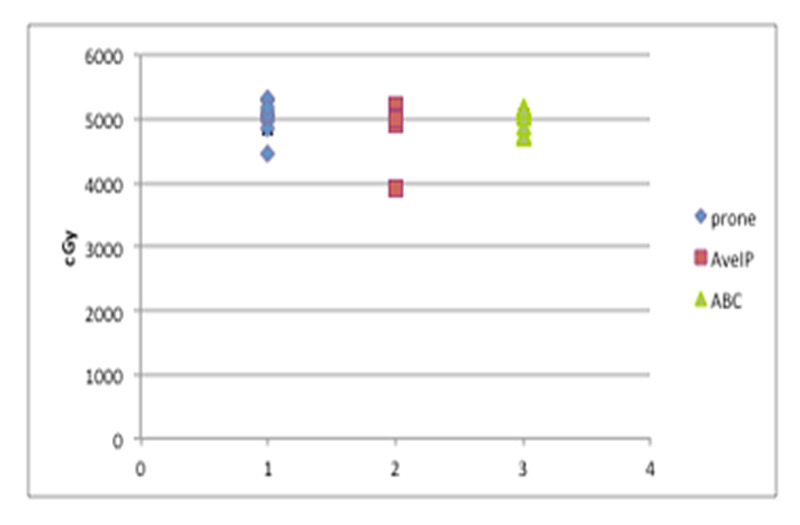
Target coverage by the simulation technique Dose to 95% of the lumpectomy PTV PTV: planning target volume

Lumpectomy PTV coverage did not significantly differ among the three plans, with 95% of the PTV lumpectomy volume covered with 100.4% of the dose in the prone plans, 98.5% in the AveIP plans, and 99.3% in the DIBH plans (p=0.7) (Figure 4).

Taken as a whole and with respect to guideline PTV coverage, prone positioning may provide an alternative acceptable option to DIBH, especially in the current pandemic. Compared to DIBH, the prone position is associated with a similar heart dose, less lung dose, and equal PTV coverage. Utilizing the prone position also further mitigates the risk of COVID-19 transmission compared with the DIBH technique.

## Discussion

In the current era of COVID-19, there has been a shift in clinical paradigms favoring strategies that mitigate clinical exposures and transmission routes [11]. As such, in the current pandemic, it is imperative to establish best practices that (1) maintain high oncologic outcomes, (2) reduce dose to OARs and respect the therapeutic ratio, (3) mitigate exposure and reduce the transmission of COVID-19, and (4) are based on data. Given the concern surrounding the use of DIBH techniques in the setting of COVID-19, we present here a dosimetric analysis comparing DIBH to other heart-sparing techniques. We demonstrate that prone positioning is superior for minimizing heart and lung radiation dose as compared with AveIp plans and superior for minimizing lung dose as compared with DIBH plans but similar with respect to cardiac parameters. Further, prone positioning has advantages in the COVID-19 era by reducing the viral transmission risk by avoiding the shared respiratory devices used for DIBH techniques. With respect to lumpectomy PTV coverage, we find no difference based on the simulation technique. Further, in patients who are not ideal candidates for prone positioning, we demonstrate that AveIP based on 4DCT can and should be utilized to determine the radiation dose to OARs to help guide clinical decision-making.

Given the data we present here, prone and DIBH plans are therefore preferable in patients where cardiac sparing is a concern such as patients receiving cardiotoxic agents or with cardiac comorbidities. Our results demonstrate that the utilization of the prone position may be equally as effective at limiting heart dose compared with DIBH plans. In the setting of the current COVID-19 pandemic, the prone position may offer a greater risk/reward benefit as compared with DIBH techniques. However, if institutions have optical surface-based imaging capabilities to couple with DIBH, this would also be a reasonable option, as it would optimize cardiac sparing and mitigate viral transmission by avoiding shared respiratory devices.

However, prone positioning may not be optimal for all patients, including those who experience anterior displacement of the heart, patient tolerance with setup, and coverage difficulty with far medial/lateral postoperative cavities. For patients who are not ideal candidates for prone positioning during the COVID-19 pandemic and with attempts to avoid DIBH, AveIP may be an alternative solution and a means to more accurately define heart and lung dose when treating with a free-breathing technique. By utilizing AveIP simulation techniques, clinicians are better able to quantify dose to OARs and are better informed when discussing with patients regarding potential long-term radiation toxicities. As such, in patients who are not ideal candidates for prone simulation, we recommend 4DCT with AveIP reconstruction to quantify dose more accurately to OARs. In patients where cardiac or lung dose is unacceptably high based on 4DCT, using alternative treatment techniques, such as free breath-hold, should be considered.

By minimizing the cardiac dose, patients can reduce their risk of long-term cardiac toxicity. Several studies have demonstrated increased cardiovascular morbidity and mortality in women with left-sided breast cancer treated with whole breast radiation therapy. A case-control study demonstrated a linear increase in the rate of major coronary events in women receiving radiation, with a 7.4% increase in risk per Gy of mean heart dose of over 2000 women [8]. No threshold mean heart dose appeared safe in this study. In addition, an increased risk was observed starting five years following the completion of treatment and persisted for at least 20 years following treatment. Therefore, long-term cardiac toxicity is of particular concern in younger breast cancer patients. Also, patients who may have other cardiac risk factors, such as obesity and diabetes, or receiving additional treatment that may predispose to heart diseases, such as anthracyclines or trastuzumab, may benefit from cardiac dose-sparing techniques. In our study, both prone and DIBH techniques resulted in a significantly lower mean heart dose as well as other dosimetric heart parameters. Rather than DIBH utilizing ABC, patients could also attempt voluntary breath-hold to attempt replicating the benefits without requiring sharing a respiratory device and thereby improving infection control [12].

Lung toxicity following radiation therapy is related to the amount of lung exposed to a certain dose of radiation. Several studies of patients with lung cancer undergoing radiation treatment show a relationship between the dose to the lung and the development of lung toxicity. Mean lung dose, V20, and V5 have all been shown to be significant predictors of radiation pneumonitis incidence. In addition, breast cancer studies show a decrease in the incidence of radiation pneumonitis with strict application of lung dose constraints such as V20 [13-15]. In our study, the prone technique resulted in consistently lower lung dosimetric parameters, including lung mean dose and lung V20, which are important predictors of pulmonary toxicity. Patients with underlying pulmonary comorbidities undergoing radiation treatment for breast cancer should therefore be treated in the prone position to achieve the maximum possible reduction in lung radiation doses.

Although these techniques can be used to prevent long-term toxicities, they can be challenging to implement in routine clinical practice. Specifically, DIBH requires the coaching of the patient by radiation therapists so the patient may learn the optimal method with which to hold their breath. This can be challenging for non-native speakers. In addition, some patients find it difficult to perform breath-hold due to potential claustrophobia, general anxiety, or underlying lung pathologies or other comorbidities. Also, this technique is more time-consuming for the patient and therapists. Prone positioning may be harder to reproduce daily and may lead to greater shifts secondary to displacement errors [6]. Due to potential difficulties in patient positioning, the improved lung dosimetry of prone plans may not be fully appreciated in practice. Therefore, if a patient has underlying lung disease, and does not set up easily in the prone position, a 4D scan with a plan based on the AveIP images might be more beneficial with a more accurately delivered lung dose.

The current analysis has several limitations. The dose prescribed is 5000 cGy and given trends toward hypofractionation, this may not represent the prescribing patterns of all radiation centers. In addition, previous publications have also commented on the impact of breast volume on dosimetry in DIBH vs prone positioning [16]. This is an important consideration in selecting best-practice approaches for cardiac-sparing techniques. Although the current analysis would benefit from an assessment of breast volume on dosimetry, we are limited by the small sample size (n=10) and are not able to report on this association. Despite these limitations, we believe our manuscript adds to the data on cardiac-sparing techniques and can help inform clinical decisions in the era of COVID-19.

## Conclusions

Given the unique circumstances that the COVID-19 pandemic has placed on patients and providers alike, we believe that this study provides a platform to explore and implement alternative techniques for sparing OARs without compromising target coverage and minimizing risks for infection. Based on our findings, we recommend prone positioning when feasible. If the prone setup is not feasible, 4DCT should be considered to obtain an accurate dosimetric representation to inform decisions regarding treatment techniques that maximize the therapeutic ratio and mitigate viral transmission. Overall, our results indicate that a personalized approach should be instituted for breast cancer patients to ensure short-term safety with regards to the pandemic and reduce late effects from radiation therapy.
